# Demographic factors associated with HIV infection between low and high prevalence areas in Nigeria, 2015

**DOI:** 10.11604/pamj.supp.2019.32.1.13330

**Published:** 2019-01-25

**Authors:** Saude Abdullahi Ibrahim, Kabir Sabitu, Aisha Abubakar, Gabrielle Poggensee, Sadiya Ibrahim, Mahammad Riyad, Adebobola Bashorun, Aminu Usman Sudawa, Baffa Sule Ibrahim, Hauwa Mohammed, Chinyere Ezeudu, Adama Ahmad Abubakar, Peter Nsubuga, Patrick Nguku

**Affiliations:** 1 Nigeria Field Epidemiology and Laboratory Training Program (NFELTP); 2 Ahmadu Bello University, Zaria, Nigeria; 3 Ministry of Education, Kano State, Nigeria; 4 Global Public Health Solutions, Atlanta, Georgia, USA

**Keywords:** HIV, HIV infection, cross-sectional study, comparative study, demography, Nigeria

## Abstract

**Introduction:**

Sub-Saharan Africa accounts for 66% of 36.7 million individuals living with HIV in 2015 with Nigeria having the second highest prevalence in Africa. The study aimed to find the prevalence and socio-demographic factors associated with HIV infection and compare these findings between high and low prevalence areas.

**Methods:**

We conducted a cross-sectional study among adults aged 15 to 49 years from March to April 2015. We administered a questionnaire to collect linked anonymous data on socio-demographic and socio-cultural characteristics and screened all respondents for HIV infection. We defined a high HIV prevalence area as area with prevalence consistently above 5% and an area with prevalence consistently below 2% as low prevalence area. We performed univariate, bivariate and logistic regration analysis to assess factors associated with HIV infection.

**Results:**

We screened and interviewed all 480 respondents. Majority 344 (71.7%) were females, mean age was 30.1 years (±7.4 years), high proportion were employed 246 (51.2%). In high HIV prevalence area, aged <30 years (Adjusted Odd Ratio (AOR) = 4.2, 95% Confidence Interval (CI) = 1.1-20.4) and being employed (AOR= 3.7, 95% CI=1.0-58.8) increased the likelihood of HIV infection. In low HIV prevalence area, lack of education (AOR=7.1, 95% CI= 0.9-32) was the only predictor of HIV infection.

**Conclusion:**

Interplay of socio-demographic factors was responsible for differences in HIV prevalence. To further decrease prevalence in low prevalence areas (below 1%), government should make universal basic education mandatory and in high prevalence areas, interventions should target the young and the employed.

## Introduction

In 2015, 36.7 million individuals were living with HIV. About 1 in 10 adults between the ages of 15 to 49 years were living with HIV/AIDS [1, 2]. Vast differences in HIV prevalence persist between countries and sub-regions [2]. The adult HIV prevalence worldwide is 1.2% [2]. Many factors accounts for variation in HIV prevalence in a population. Geographical differences as well as demographic factors are important risk factors for HIV infection. Poor urban duellers are more likely to be HIV positive as shown by demographic and health survey in Malawi in 2004, were HIV prevalence is more among females and urban duellers [3]. A study among severely malnourished children at nutrition centre in Malawi showed that HIV prevalence is highest among urban children [4]. Different cultural practices among the population affect HIV prevalence rate. Having more than one concurrent sexual partner is a major factor for the variations in prevalence as demonstrated in an ecological analysis in Africa by Prudden et al [5, 6]. Areas that practice early marriage exposed the population to early age at sexual debut, this increased adolescent fertility rate, which are predisposing factors for HIV infection [7, 8]. Availability of physician may improve diagnosis and health care services delivery, this will in turn increase the diagnosis of HIV and HIV prevalence will be higher, compared to areas with low physician density [9]. A randomised trial in Africa showed that treatment of sexually transmitted infection reduces the incidence of HIV infection [10]. HIV prevalence also varies among different geographic region with different socio-cultural practices, unemployment and people refusing to participate in a HIV survey are more likely to be HIV positive, while an area with a high proportion of Muslims have low prevalence [11]. The sub-Saharan region is heavily affected by HIV/AIDS, and accounts for about 66% of the people living with HIV worldwide in 2015 [1]. The Western and Southern part of sub-Saharan Africa are the worst affected by HIV/AIDS (the so-called “AIDS belt”) [1]. This belt consists of 16 countries and constitutes about 4% of the world's population. The belt account for more than 50% of HIV infection worldwide [2]. Nigeria is one of the country in the AIDS belt and has the second highest HIV prevalence and a national prevalence trend that varies from 1.8% in 1991, 4.1% in 2010 and 3.4% in 2012 [1, 12]. In Nigeria, from 2001 to 2008, the prevalence showed a consistently falling trends in five states, a consistently high prevalence in 12 States including Federal Capital Territory (FCT), and consistently low prevalence in five States including Jigawa State [13, 14]. In Nigeria, the first case of AIDS was first reported in 1986 [15]. Since then, the national prevalence trend varied from 1.8% in 1991 to 4.0% in 2010, with the peak in 2001, 5.8% [16]. The HIV prevalence also varied by states and different at-risk groups. Observation of HIV prevalence trends of states from 2001 to 2008 revealed a consistently falling pattern in six states (Plateau, Bauchi, Ekiti, Gombe, Zamfara and Abia) [17]. FCT and some states, (Kaduna, Akwa-Ibom, Cross River, Rivers, Benue, Kogi, Nasarawa, Niger, Taraba, Adamawa and Enugu), continue to have a consistently high HIV prevalence, 5.0% and above. From 2003 to 2008, few states, (i.e., Ekiti, Osun, Ogun, Oyo and Jigawa), have had a consistently low prevalence, 2.0% or less [16]. To our knowledge, there is no published work done in Nigeria that takes into consideration a cosmopolitan society like FCT or relatively homogeneous community like Jigawa State and how these differences may affect the HIV prevalence. This study therefore aimed to compare demographic factors associated with HIV infection in Jigawa State and FCT.

## Methods

### Study area

#### Jigawa State

**Figure 1:**
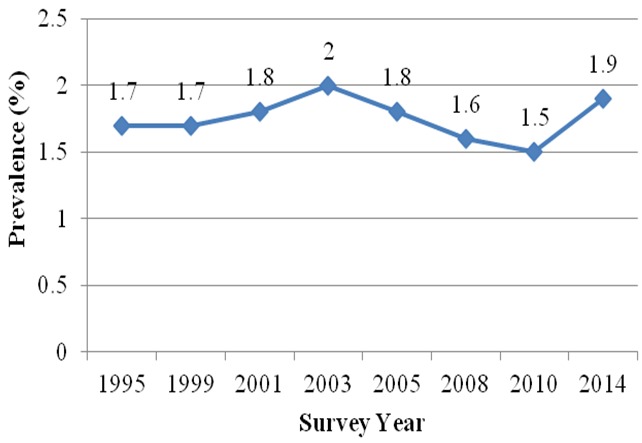
Prevalence of HIV infection in Jigawa State, 1999 to 2014

Jigawa State has a population of about 3.6 million (as projected from 2006 census), 90% live in the rural area and 50.8% are males. Hausa-Fulani and Mangawa are the main ethnic groups [18]. The main occupation of the inhabitants is farming. The Antenatal care (ANC) HIV seroprevalence sentinel survey was conducted first in 1995 and the state prevalence has never been above 2%, from 1995 to 2014 [16], (Figure 1).

#### Federal capital territory

**Figure 2:**
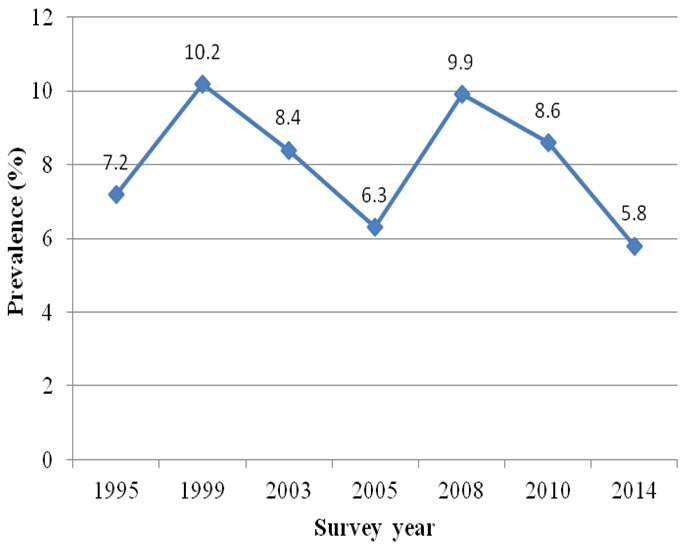
Prevalence of HIV infection in FCT, 1995 to 2014

FCT is the administrative state of the federation, it has a population of about 3,028,807 (2006 Census projection). The main indigenous ethnic groups are the Kanuri, Bassa, Gade, Gwandara, Koro, Ganagana [19]. People living in urban part of FCT are mostly civil savant or engage business activities while those living in rural part are mostly famers. FCT became part of the national HIV seroprevalence sentinel survey only after the third round of the survey and the HIV prevalence has never been below 5.8% [16] (Figure 2).

**Study design:** the study design was a cross-sectional study carried out from February to April 2015.

**Study population:** were adult men and women aged 15-49 years.

**Inclusion criteria:** adults aged 15 to 49 years who were permanent residents or had resided for > 5 years in either state and consented for biological part of the survey. For respondents < 18 years, we obtained consent from their parents, guardians or husbands.

**Exclusion criteria:** adults who were not permanent residents or had resided for < 5 years in either state and respondents that did not consent for the biological part of the survey (HIV counselling and testing).

**Sample size determination:** we used the formula for comparing two proportions to calculate the sample size for the population-based survey [20].

$$\text{m'=}{\left[\frac{C_{\frac{\alpha }{2}}\sqrt{(r+1)P_{0}Q}-C_{\left(1-\beta \right)}\sqrt{rP_{1}Q_{1}+P_{2}Q_{2}}}{r{\left(P_{2}-P_{1}\right)}^{2}}\right]}^{2}$$

$$m=m'\left[1+\frac{\sqrt{1+2(r+1)}}{4\text{m'}r(p_{2}p_{1})}\right]$$

Where: m = n_1_ = size of sample from FCT, n_2_ = size of sample in Jigawa State.

P_1_ = prevalence of HIV infection in FCT (2010) = 0.086

Q_1_ = 1 -P_1_ = 0.914

P_2_ = prevalence of HIV infection in Jigawa (2010) = 0.015

Q_2_ = 1 -P_2_ = 0.985

r = ratio of the two states population = n_1_ / n_2_ = 64.7% / 35.3% = 1.53

P_0_ = P_1_ - P_2_ = 0.086 - 0.015 = 0.071

Q_0_ = 1 -P_0_ = 1 - 0.071 = 0.929

α = “Significance” = 0.05

β = chance of not detecting a difference = 0.2 1 - β = power = 0.08

1 - α = 0.95, Cα/2 = 1.960

1 -β = 0.80, C1-β = 0.842

After substituting the above figures, m' was found to be 98.69 and thus, m = n_1_ = 84.03

n_2_ = r x m = 1.83 x 84 = 154

The total minimum sample size was 154 + 84 = 238 and by multiplying the sample by a factor of two in-order to increase the sample size, total sample size was 476.

For FCT, the sample size was 168. For Jigawa State, the sample size was 308. The sample size was different in the two states, because prevalence rate was used in the calculation, which was different (Jigawa = 1.5 % and FCT = 8.6%).

**Sampling technique:** we used stratified simple random sampling technique in both states.

**Stage 1:** we selected three Local Government Areas (LGA) or Area councils from each state by simple random sampling.

**Stage 2:** we selected one rural and one urban ward by simple random sampling (SRS) from each LGA or area council. An urban ward is defined by the National Population Commission as a community that has a population of > 20,000 people, while a rural ward is a community that has a population of < 20,000 people [21].

**Stage 3:** from the list of all settlements in the ward, we randomly selected one settlement.

**Stage 4:** we selected equal numbers of households from each settlement, and we selected one eligible individual from each household, we selected subsequent eligible individuals by SRS.

**Data collection tools:** we used semi-structured interviewer-administered questionnaire adapted and modified from Nigeria Demographic and Health Survey, 2013 [22]. We pretested the questionnaire in Gwagwalada area council (a non-selected area council), on 30 respondents, which were not part of the study sample. The questionnaire contained sections that obtained data on socio-demographic and socio-cultural risk factors.

**Training of field workers:** we recruited graduate or National Youth Service Corps (NYSC) members as interviewers and trained three laboratory scientists/ technologists as counsellors and testers. We conducted 2-day training on the survey's objectives in each state.

**HIV testing algorithm:** we used three serial testing algorithm based on the recommendation of the Federal Ministry of Health [23], using Stat-pack as the first line, Uni-Gold as confirmatory and Sure-check as a tiebreaker.

**Data management:** we analysed all data using Epi-Info version 7(US Centres for Disease Control and Prevention), SPSS (IBM Corporation) and Microsoft Excel software and we accepted a p-value of <0.05 as statistically significant. We calculated means and standard deviations for continuous variables and proportions and 95% confidence intervals for categorical variables. We conducted bivariate analysis using HIV seropositivity as the dependent variable and demographic risk factors as independent variables. We calculated odds ratios (OR), and we determined statistical significance using chi-square or Fisher's test as appropriate, along with p-values.

**Ethical consideration:** we obtained approvals from the ethics committee of the two States Governments and Ahmadu Bello University Teaching Hospital. We obtained clearance from Jigawa states and the Federal capital territory governments, district heads and heads of households. We collected unlinked anonymous data and written informed consent from all respondents, participants were informed that participation is entirely voluntary and they can withdraw at any point of the study. We obtained the informed consent by reading a clear and concise summary of the study to each participant, emphasising the role of the participants ensuring the confidentiality of all their information. Each participant signed/ thumb printed the informed consent forms and a copy was giving to each participant.

## Results

We interviewed a total of 480 respondents, 168 from FCT and 312 from Jigawa State.

**Socio-demographics:** the majority 344 (71.7%) of the respondents were females. The mean age of respondents was 30 years [Standard deviation (SD) ± 7 years] in FCT and 30 years (SD ± 8 years) in Jigawa. Higher proportions of respondents were unemployed in FCT 102 (61%) in contrast to Jigawa, were the majority of respondents were employed 180 (58%), 93 (55%) were Muslims in FCT while in Jigawa, 308 (98.7%) were Muslims. The majority of respondents, 163 (52.2%), had formal, Qur'anic/Islamiya educations in Jigawa while 105 (62.5%) had only formal education in FCT.

**HIV prevalence:** we found HIV prevalence in FCT of 6.5%(11/168) and in Jigawa was 1.9% (6/306).

**Bivariate analysis:** factors found to be associated with HIV infection in the high prevalence state (FCT) were age > 30 years (OR = 3.5, 95% CI = 3.5-12.3), having employment (OR = 4.6, 95% CI = 1.2-17.8). However, in the low prevalence state (Jigawa), factors associated with HIV infection was being a Christian (OR = 15.1, 95% CI = 1.4-160). Lack of education (both formal and Qur'anic) (OR = 8.9, 95% CI = 1.7 - 45.9) and not having Qur'anic education (OR = 15.1, 95% CI = 1.3 - 172) were also associated with HIV infection in the low prevalence state (Table 1).

**Table 1:** Socio-demographic factors and HIV prevalence among respondents in FCT and Jigawa State, April, 2015

Variable	All respondents: n (%)	FCT: n (%)	Jigawa: n (%)
**Sex**			
Male	136.0 (28.3)	12.0 (7.0)	124.0 (40.0)
Female	344.0 (71.1)	156.0 (93)	188.0 (60.0)
Mean age in years (standard deviation)	30.1 (7.4 years)	30.0 (7.0 years)	30.0 (8.0 years)
**Employment status**			
Employed	246.0 (51.2)	66.0 (39.0)	180.0 (58.0)
Unemployed	234.0 (48.8)	102.0 (61.0)	132.0 (42.0)
Religion			
Muslim	400.0 (83.3)	93.0 (55.0)	308.0 (98.7)
Christian	80.0 (16.7)	75.0 (45.0)	4.0 (1.3)
**Educational status**			
None	60.0 (12.5)	26.0 (15.5)	34.0 (10.9)
Qur’anic/Islamiya	112.0 (23.3)	31.0 (18.5)	81.0 (26.0)
Formal & Quranic/Islamiya	169.0 (35.2)	6.0 (3.5)	163.0 (52.2)
Formal only	139.0 (29.0)	105.0 (62.5)	34.0 (10.9)
**Marital status**			
Single	61.0 (12.7)	5.0 (3.0)	56.0 (18.0)
Married	389.0 (81.0)	153 (91.1)	236.0 (75.6)
Divorced/Widowed/Separated	30.0 (6.2)	10.0 (6.4)	20.0 (6.4)
**HIV test Result**			
Positive	17.0 (3.5)	11.0 (6.5)	6.0 (1.9)

**Multivariate analysis:** factors that were responsible for persistently high HIV prevalence were having employment (adjusted Odds Ratio (AOR) = 5.1, p-value = 0.02) and age less than 30 years (AOR = 4.4, p-value = 0.04) (Table 2). However, in Jigawa (low prevalence state), the only factor associated with HIV seropositivity was a lack of education (AOR = 7.1, p-Value =0.01) (Table 3).

**Table 2:** Predictors of HIV infection in FCT, April 2015

Variable	HIV Positive: n (%)	HIV Negative: n (%)	OR(95 % CI)	p-value	AOR(95 % CI)	p-value
**Age group(years)**						
< 30	5.0 (14.0)	30.0 (86.0)	3.5 (1.0-12.3)	0.04	4.2 (1.1-20.4)	0.04
> 30	6.0 (5.0)	127.0 (96.0)				
**Religion**						
Christian	6.0 (8.0)	69.0 (92.0)	1.5 (0.4-5.2)	0.5	0.5 (0.05-4.3)	0.5
Islam	5 (4)	88.0 (95.0)				
**Occupation**						0.05
Employed	8.0 (12.0)	58.0 (88.0)	4.6 (1.2-17.8)	0.02	3.7 (1.0-58.8)	
Unemployed	3.0 (3.0)	99.0 (97.0)				
**Education**						
Uneducated	2.0 (8.0)	24.0 (92.0)	1.2 (0.3-6.1)	0.8		
Educated	9.0 (6.0)	133.0 (94.0)				
**Qur’anic education**						
No	7.0 (7.0)	98.0 (93.0)	1.4 (0.01-2.9)	0.2	1.4 (0.01-2.9)	0.3
Yes	2.0 (5.0)	35.0 (95.0)				

**Table 3:** Predictors of HIV infection in Jigawa state, April 2015

Variable	HIV Positive n (%)	HIV Negative n (%)	OR(95 % CI)	p-value	AOR(95 % CI)	p-value
Age group(years)						
<30	1.0 (0.4)	233.0 (99.6)	0.06 (0.01-0.5)	< 0.01	7.6 (0.0-7.4)	1.0
>30	5.0 (6.4)	73.0 (94.0)				
Religion						
Christian	1.0 (20.0)	4.0 (80.0)	15.1 (1.4-160.0)	< 0.01	5.9 (0.0-21.4)	1.0
Islam	5.0 (2.0)	302,0 (98.0)				
Occupation						
Employed	1.0 (1.0)	179.0 (99.0)	0.14 (0.02-1.2)	0.04	3.6 (0.3-32)	0.06
Unemployed	5.0 (4.0)	127.0 (96.0)				
Education						
Uneducated	3.0 (8.8)	31 (91.2)	8.9 (1.7-45.9)	< 0.01	7.1 (0.9-16.8	0.01
Educated	3.0 (1.0)	275 (99)				
Qur’anic education						
No	2.0 (6.0)	32.0 (94.0)	15.1 (1.3-172.0)	< 0.01	2.0 (0.0-3.1)	1.0
Yes	1.0 (0.4)	243.0 (99.6)				

## Discussion

We explored socio-demographic factors associated with differences in HIV prevalence observed between two different prevalence areas. In high prevalence area, we found unemployment and age less than 30 years were the determinant of HIV infection, while in low HIV prevalence area, lack of education was the only predictor of HIV infection. Age is an important factor that determines HIV infection. Respondents below 30 years were more likely to be HIV infected in the high prevalence area, which may mean this group were more sexually active and more prone to risky sexual behaviours compared to older age group. The difference found among different age groups in high prevalence area in this study may be from differences in cultural practices that prevent risky behaviours in Jigawa State such as polygamous family setting and high level of Qur'anic education [24, 25]. These factors, coupled with the high living standard in FCT, could imply that people may be engaged in risky behaviours, such as prostitution, to cope. This finding is in contrast to a review in Nigeria and National reproductive and health survey 2012 (plus II), which showed a high prevalence among 35-39 years age group [16]. A similar finding was seen in a study from South Africa were a higher prevalence of HIV infection was seen among young people [26].

This study showed that those who were employed were less likely to have HIV infection in low prevalence state, employment allows a person to be financially independent, and therefore able to afford other necessities of life such as education, food and access to health services. Having employment may also allow them to afford a certain standard of living, such as alcoholism, promiscuity, that may increase their chances of having HIV infection. This finding is contrary to a study in South Africa were unemployment and low level of education were found to be associated with HIV infection [27], Education attainment allows an individual or group of people to have a clear perspective about life issues including disease prevention. From our study, lack of education increases the likelihood of having HIV infection which is statistically significant in low prevalence state. Findings from a population based survey in Nigeria showed that respondents with higher educational level were more likely to be HIV infected than those with no formal education [28]. This finding is similar to a study in Zimbabwe, which showed that HIV prevalence fell steeply among more educated group [29]. A hospital-based study in India on socio-demographic factors associated with HIV infection showed that low educational attainment was associated with HIV infection [30]. A study of educational attainment and HIV-1 infection in developing countries shows that higher educational attainment is associated with a greater risk of HIV infection with changing pattern towards a greater burden among less educated groups, but a study in Thailand, showed that those with more schooling remain at lower risk of HIV infection [31].

### Limitation

Some respondents were sceptical in participating but adequate information about the aims and objectives of the study was given.

## Conclusion

To successfully decrease and control HIV infection in different prevalence areas, government interventions should be specifically tailored to specific population groups. The government should make universal basic education mandatory in order to further reduce HIV prevalence in low prevalence states. For control in the high prevalence area, intervention programs should be customised targeting young and the employed.

### What is known about this topic

- HIV infection is a pandemic disease;
- Prevalence of HIV infection varies in different geographic region;
- Multiple sexual partner is a risk factor for HIV infection.

### What this study adds

- Socio-demographic factors are responsible the differences in HIV prevalence at different geographic areas;
- Illiteracy is responsible for HIV infection in low prevalence area;
- Employment and being less than 30 years old are responsible for HIV infection in high prevalence area.

## Competing interests

The authors declare no competing interests.
